# Impulse Response of the Elasto-Hydrodynamic Lubrication Film of a Rolling Bearing to Dynamic Excitation of a Flat Belt Drive

**DOI:** 10.3390/ma13204533

**Published:** 2020-10-13

**Authors:** Pavel Adamčík, Zuzana Murčinková

**Affiliations:** 1 Technická Diagnostika, Ltd., Jilemnického 3, 080 01 Prešov, Slovakia; p.adamcik@diagnostika.sk; 2 Department of Design and Monitoring of Technical Systems, Faculty of Manufacturing Technologies with seat in Prešov, Technical University of Košice, Bayerova 1, 080 01 Prešov, Slovakia

**Keywords:** impulse response, beating excitation cycle, elasto-hydrodynamic lubrication film, bearing life, fatigue damage

## Abstract

The impulse response of a rolling bearing and its principal component, the elasto-hydrodynamic lubrication film (EHDL), are analysed. When measuring the vibrations of bearings, we observed that the impulse response was mostly caused by defects (fatigue damage) on the raceways and/or rolling elements. However, this phenomenon can also occur in new defect-free roller bearings, where it is not commonly expected. This study presents an experiment that identifies the conditions of dynamic excitation for the impulse response of the EHDL, the source of which is not defects, but the EHDL itself. The EHDL responds in the form of impulses in case the velocity of its radial deformation is too fast. This is an unfavourable phenomenon that significantly shortens the service life of bearings. To analyse the dynamic excitation conditions, a testing bench at speeds up to 135,000 rpm with a flat belt drive was used. The testing bench enabled the formation of the so-called beat excitation from two harmonic excitation forces close in rotational frequency. The subject of this study is a defect-free high-speed double-row angular contact ball bearing used in the textile industry. We also present other physical conditions for the occurrence of undesired impulse responses that are caused by the EHDL.

## 1. Introduction

In production, when rotating machines operate, vibrations occur. Rolling bearings are one of the most important components of mechanical systems. Highly loaded components for the transmission of mechanical power, especially rolling bearings and gears, exhibit a complex measured dynamic signal. The signal is characterised by a complex amplitude modulation or a pulsed (nonsinusoidal) signal shape. The impulse shape of the signal is most often a symptom of an incomplete lubrication film in the formation of a partial metal contact of surfaces, while typical symptoms of fatigue damage arise, such as subsurface cracks and subsequent surface defects of contact surfaces (pitting).

The presented research is mainly focussed on the analysis of impulses that occur in the measured vibration signal and are not excited by the passage of the rolling elements through defects such as cracks, pits, and spalls. However, many researchers have developed analytical and numerical models that have experimentally focussed on that “shock” excitation on defects.

**Figure 1:**
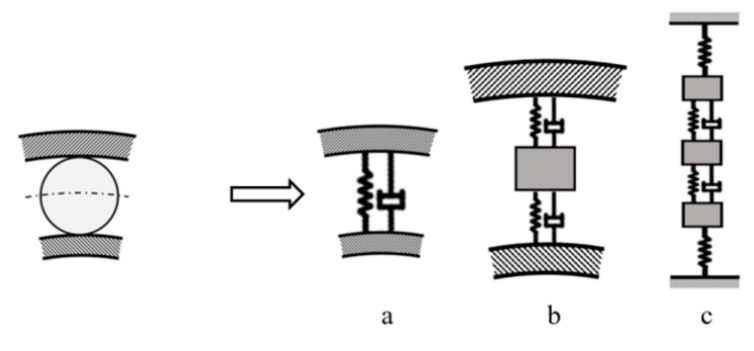
Bearing dynamics models, (**a**) spring-damper system without single lubrication film, (**b**) involved lubrication film with nonlinear damping, (**c**) involved bearing rings.

The bearing dynamic model can be quite complex, according to Figure 1, although the drive, bearing housing, and supports are not involved.

According to [1,2], the greatest simplification of the dynamic bearing model is the spring–damper system (Figure 1a), in which a nonlinear contact where the lubrication film is not a single element of the dynamic system is considered. According to [3] (Figure 1b), a lubrication film can be treated as a nonlinear spring with nonlinear damping at the ball–raceway contact. The dynamic bearing model in Figure 1c, according to [4], has three degrees of freedom and includes the stiffness of the outer and inner rings. The bearing housing is considered to be rigid.

The selected dynamic models in Figure 1 are the starting points for the mathematical derivation of the behaviour and response of a dynamic rolling bearing system. The developing dynamic models of rolling bearings have specific prerequisites for the prediction and simulation of the dynamic response [5,6,7,8] and are designed mainly for low speeds; consequently, their accuracy decreases under high-speed conditions. A few dynamic models are suitable for high velocities and consider the effect of centrifugal force, gyroscopic moment, and time-varying contact angles [9,10]. Dynamic models with impulse response include various factors such as nonuniform surface waviness [11], defects with different edge shapes [12], and defect size determination [13]. An overview of models is presented in [14,15]. The disadvantage of many models is that they do not consider the lubrication film or take into account its contact damping [16].

However, the limitation of bearing dynamic models lies mainly in the area of not taking into account the elasto-hydrodynamic effects of the lubrication film. The mechanical and physical material properties of elastic deformable bodies (rolling raceways and rolling elements) can be described in terms of linear as well as nonlinear dependence. The contact of the bodies is a boundary nonlinearity. However, the lubrication film has fluid mechanical–physical properties, which are fundamentally different from the properties of solids; thus, the response of the lubrication film is specific.

Metal-to-metal contact without considering the lubrication film was an approach used in the first half of the 20th century but has limited usage today as the lubrication film is known to cause significant effects. The lubrication film fulfils an elastic and, at the same time, a damping function and is an important component of the contact of the bodies that determine their service life. The thickness of the lubrication film is a function of speed, load, geometry, material, and lubricant properties. The lubrication film thickness initially increases with speed, but at a certain critical speed, the thickness of the film begins to decrease with increasing speed in the so-called starved regime [17]. The increase in speed results in an unstable response of the system [3]. Similarly, the starvation regime can occur not only at excessive speeds but also at high lubricant viscosities or small amounts in contact.

In contrast to the above-mentioned local fault excitation models with the presence of the impulse response of a rolling bearing in the measured vibration signal, our case describes the impulse response of an elasto-hydrodynamic lubrication film (EHDL) resulting from non-Newtonian physical properties of the lubrication film. The vibration signal was measured on an angular contact ball bearing designed as a high-speed textile rotor. During the test, the measured bearings had a speed of 135,000 rpm (i.e., a rotation frequency of 2250 Hz). We tested a new defect-free bearing in which a decrease in the quality of the rolling race microgeometry (roughness and waviness) was not found, neither before nor after the experiment. There was no limited frictional regime during the bearing operation. The contact surfaces were completely separated by a continuous, load-carrying lubrication film. There was no fatigue damage (e.g., no pitting) on the contact raceways of the bearing. The aim of the study was to determine the conditions of dynamic excitation, when the dynamic system of the rolling bearing, including the EHDL, behaves nonlinearly and produces an impulse response (pulse signal shape) that is not caused by defects.

## 2. Materials and Methods

**Figure 2:**
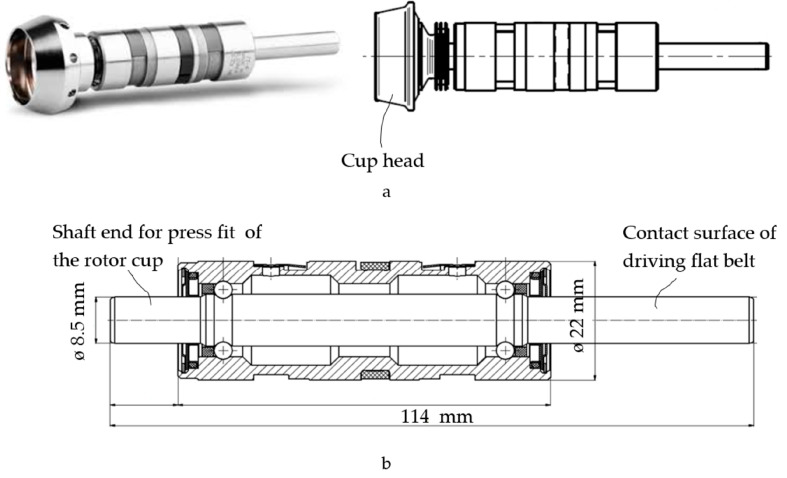
Rotor bearing (**a**) and section (**b**).

To observe the phenomenon of impulse response of the EHDL without influence of the environment, we used laboratory conditions. We used new, undamaged, defect-free textile rotor bearings, which are double-row angular contact ball bearings (Figure 2).

The rotor bearings were made of standard components that met all prescribed dimensional and geometric tolerances. The bearings did not exhibit any deviation from the prescribed quality of the raceway microgeometry. The bearings were tested on a test bench after a 24 h run-in.

The manufacturing of textile rotor bearing is not standard production. Basically, the rotor bearings are special products characterised by specific materials and a high precision of production.

The rotor bearing shaft is made of a high-nitrogen (0.3–0.5%) martensitic cold-work tool steel (carbon content 0.25–0.35%) with a fine-grained homogeneous structure named Cronidur that is characterised by high hardness (>58 HRC, Rockwell scale), high strength (2150 MPa), and high corrosion resistance under different temperatures even in aggressive media and outstanding tribological properties. The content of chromium is 14–16%. The bearing bush is made of standard bearing steel 100Cr6. The material of the bearing cage is fibre-reinforced polyamide for high-speed operation that has the ability of low temperature rise. The rolling balls are made of non-oxide ceramics: silicon nitride Si_3_N_4_. A summary of the used materials is in Table 1.

The lubricant used in the experiment is industrial lubricating grease for high-speeds, e.g., Klüberspeed BF 72-22, Klüber Lubrication Australia, Melbourne, Australia. The mentioned high-performance lubricant developed for extremely high speeds is a special grease with a synthetic base oil and polyurea thickener. The viscosity–temperature characteristics are suitable for a wide range of speeds and temperatures. The chemical composition provided by producer is as follows: synthetic hydrocarbon oil, ester oil, polyurea.

Table 2 shows the precision grade of a high-speed rotor bearing that is 5–10 times higher than the standard production of bearings.

### 2.1. Testing Bench and Measuring Chain

**Figure 3:**
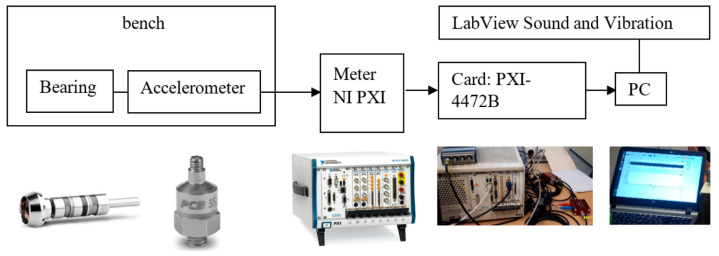
Measuring chain components.

**Figure 4:**
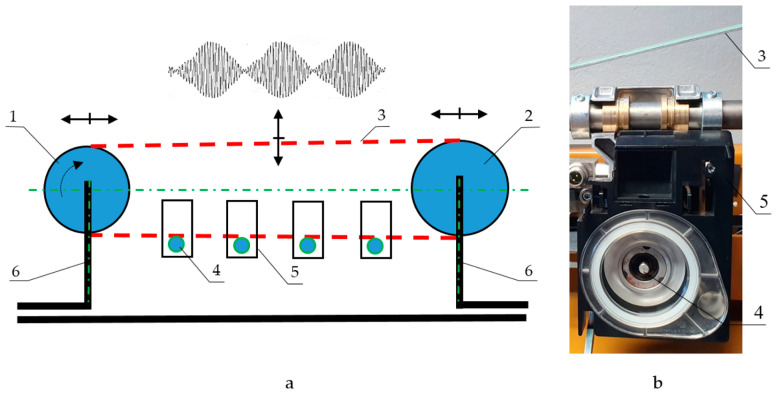
Testing bench: (**a**) schema; (**b**) detail; 1—pulley with *D*_1_ = 240 mm, 2—pulley with *D*_2_ = 250 mm, 3—flat belt, 4—rotor bearings, 5—bearing housing, 6—beam structure.

The measurements were performed on a testing bench (Figure 3 and Figure 4).

To measure and collect the dynamic data (of absolute vibration, as recommended by ISO standard 10816-3), the following measuring chain in Figure 3 and its components were used: an accelerometer (PCB model 352A60, PCB Piezotronics, Depew, NY, USA), with a frequency range up to 65 kHz and a sensitivity of 10 mV/g; a National Instruments PXI metre, National Instruments Corporation, Austin, USA; and LabView Sound and Vibration Toolkit software with a PXI-4472B PXI Sound and Vibration Module Meter, National Instruments Corporation, Austin, TX, USA.

A testing bench with a flat belt was used to test the bearing. For the dynamic radial load of the bearing, the so-called beat excitation was applied as the sum of two harmonic vibrational components that were close in frequency. This load was excited by the oscillation of the flat drive belt, which contacts the end of the shaft.

The testing bench, the scheme, and details are shown in Figure 4. The bench was emplaced as a section of a textile machine for testing and research. Pulleys 1 and 2 have similar diameters, (i.e., 240 and 250 mm, respectively) to achieve close rotational frequencies. Belt 3 contacts the shafts of rotor bearing 4 with a maximum rotational speed of 135,000 rpm. Each rotor bearing is mounted in the bearing housing and ensures against axial displacement. An accelerometer was fixed to the bearing housing 5. To cause variation in the tension of belt 3 and thus increase the recorded transverse vibrations, the speed of the pulleys was chosen close to the natural frequency of beam structure 6, which is in the range of 70–80 Hz.

### 2.2. The Loading Beat Excitation

**Figure 5:**
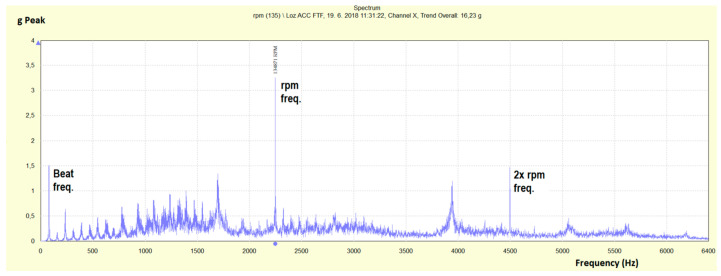
Fast Fourier transform (FFT) spectrum of acceleration (g Peak) at a rotational speed of 134,870 rpm and a band up to 6400 Hz.

The testing bench represents a mechanical system whose fast Fourier transform (FFT) spectrum of acceleration in the range of 0–6400 Hz at a rotational speed of 134,870 rpm is shown in Figure 5.

Frequency peaks of the load beat frequency (Beat freq.) and the rotor-bearing peak (rpm freq., 2248 Hz) are marked among the frequency peaks of the testing bench components.

**Figure 6:**
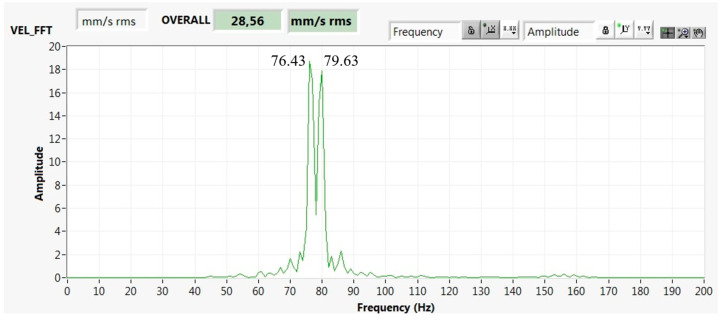
FFT spectrum of velocity (mm/s Peak), with two excitation components generating the loading beat excitation and drive pulleys rotational frequencies of 76.43 and 79.63 Hz.

The bearing was radially subjected to two independent harmonic excitation forces with a small rotational frequency difference, as shown in Figure 6.

The driven and driving pulleys of the belt drive had rotational frequencies *f*_1_ = 76.43 and *f*_2_ = 79.63 Hz; that is, Δ*f* = 3.2 Hz. The driven pulley rotational speed was *n*_1_ = 4585.8 rpm, and the driving pulley rotational speed was *n*_2_ = 4777.8 rpm. The angular velocities were *ω*_1_ = 480.22 rad/s and *ω*_2_ = 500.33 rad/s. In our case, $f_{1}-f_{2}$ <<$\;f_{1}+f_{2}$.

The vibration equation of motion in scalar form for this case is as shown in Equation (1):

(1) $$m\ddot{x}+b\dot{x}+kx=F_{1}\sin\omega_{1}t+F_{2}\sin\omega_{2}t$$

By using the superimposing principle, the steady forced oscillation of the system is as shown in Equation (2):

(2) $$x=x_{1}+x_{2}=A_{1}\sin\left(\omega_{1}t-\varphi_{1}\right)+A_{2}\sin\left(\omega_{2}t-\varphi_{2}\right)$$

where *A*_1_ and *A*_2_ are the amplitudes of the two oscillating components, *ω*_1_ and *ω*_2_ are the angular velocities*,* and *φ*_1_ and *φ*_2_ are the initial phases. The resulting solution of Equation (1) describing the steady forced oscillation of the system is as shown in Equation (3):

(3) $$x=\sqrt{B_{1}^{2}+B_{2}^{2}}\sin\left(\frac{\omega_{1}+\omega_{2}}{2}t-\frac{\varphi_{1}+\varphi_{2}}{2}+\varphi *\right)$$

where $tg\varphi *=\frac{B_{1}}{B_{2}}$ with Equation (4):

(4) $$B_{1}=\left(A_{1}+A_{2}\right)\cos\left(\frac{\omega_{1}-\omega_{2}}{2}t-\frac{\varphi_{1}-\varphi_{2}}{2}\right)\;B_{2}=\left(A_{1}-A_{2}\right)\sin\left(\frac{\omega_{1}-\omega_{2}}{2}t-\frac{\varphi_{1}-\varphi_{2}}{2}\right)$$

**Figure 7:**
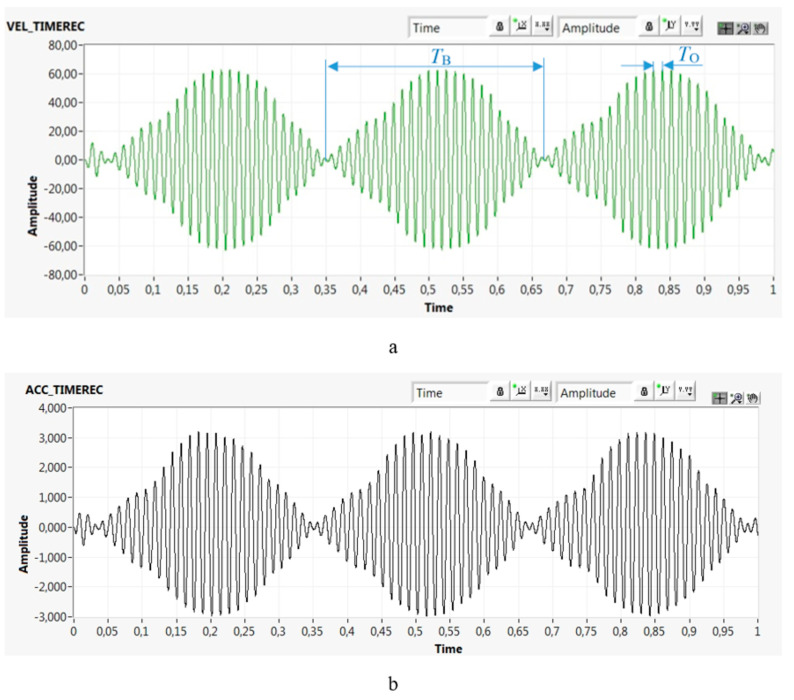
Beating cycles in the time domain over 1 s: (**a**) velocity (mm/s) vs. time (s); (**b**) acceleration (g) vs. time (s).

Figure 7 shows the time domain record of velocity (Figure 7a) and acceleration (Figure 7b) of beating cycles generated by superimposing two harmonic motions from the driving and driven pulleys after the amplitude modulation.

The amplitude of the resulting motion of the transverse belt vibrations changes periodically in time and is not a simple sinusoid. The amplitude varies between the two values. Beats are formed with a beating period *T*_B_. A sinusoid is the envelope of the time record of the oscillations. As the flat belt is in contact with the bearing shaft, the bearing is radially loaded by beating excitation cycles in the acceleration range of ±3.2g (peak).

The advantage of beat excitation is that it integrates various velocities and accelerations. Therefore, it provides an advantage because we do not have to simulate individual operating modes that generate certain velocities (accelerations) at different rotational frequencies. For example, such different operating modes can be achieved by adding different large imbalances onto the driving pulley and analysing the measured response. However, using the beat excitation, we have a mechanical system loaded cyclically with repeated increases and decreases in amplitudes and repeated changes in velocity (acceleration). Moreover, it is possible to change the beating and oscillation periods by changing the values of the rotational frequencies *f*_1_ and *f*_2_ according to Equation (5):

(5) $$T_{B}=\frac{1}{\left|f_{1}-f_{2}\right|}=\frac{1}{3.2\;\mathrm{Hz}}=0.3125\;\;s$$

and Equation (6):

(6) $$T_{O}=\frac{2}{f_{1}+f_{2}}=\frac{2}{156.06\;\mathrm{Hz}}=0.0128\;\;s$$

where *T*_B_ is the beat period and *T*_O_ is the oscillation period. The calculated values are for the beating excitation in Figure 7.

## 3. Results of Experiment

**Figure 8:**
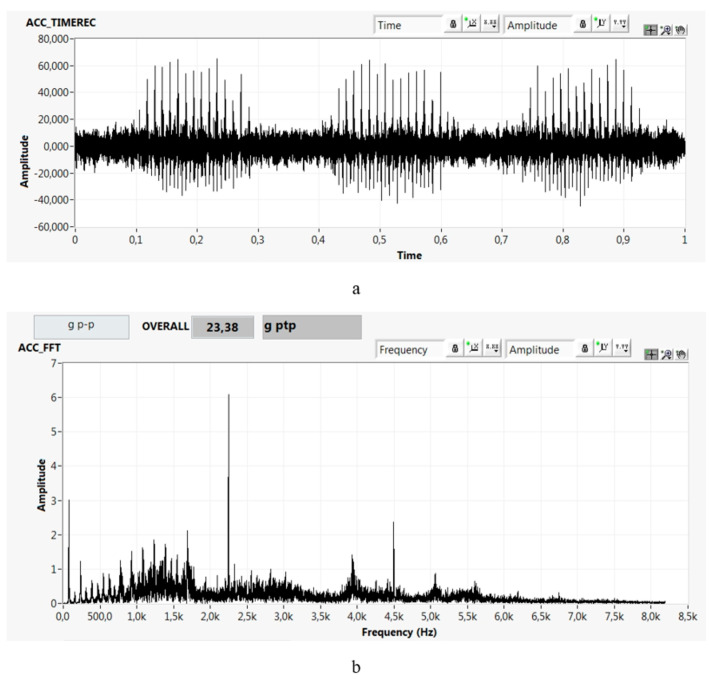
Impulse response of the bearing in the radial direction: (**a**) acceleration (g) vs. time (s) and (**b**) its FFT spectrum, acceleration FFT (g peak to peak) vs. frequency (Hz).

Prior to the experiment presented in the paper, the measurements and observations of impulse response were previously performed at approximately 3000 high-speed rotor bearings. For the needs of the experiment in the paper, the impulse response was measured for ten rotor bearings. Under the conditions described in Section 2, time records of the impulse response were obtained. One of the recorded impulse responses is in Figure 8a with the FFT spectrum shown in Figure 8b.

The size of the impulses was up to 62 g, which is almost a factor of 10 higher than the limit for safe operation (up to 7 g). This means that the system raceway–rolling element–lubrication film was subjected to extreme loads. The duration of documented impulses was 3–4 ms. The impulses were high frequency, as shown in Figure 8b, with high energy. The narrow frequency peak of 2250 Hz in Figure 8b is the rotational frequency of the bearing. Other frequency peaks are wide with sidebands.

By comparing Figure 7 and Figure 8a, it can be seen that the extreme impulses do not occur throughout the time record. Therefore, the measured impulses are not impulses caused by passing the rolling elements through defects (because the bearing is defect free). Impulses begin to arise and disappear in certain sections. In the documented case, no significant impulses were seen in cyclically repeating time periods—for example, between 0.28 and 0.44 s (Figure 8a). In contrast, the impulses were very significant in cyclically repeating time periods—for example, between 0.12 and 0.28 s (Figure 8a). The repeating sections with and without significant impulses are related to the beat excitation characterised by a cyclical variation in velocity and acceleration. During one beating period *T*_B_, the amplitudes varied in a wide range from 4 to 60 mm/s (Figure 7a) in the short individual oscillation periods (*T*_O_ = 12.8 ms; Equation (6)). Significant impulses arose only from certain values of the change in velocity (acceleration) of the excitation. In this case, the velocity was ≈30 mm/s and the acceleration was 1.5 g (Figure 7a) in that oscillating period *T*_O_. The impulses in Figure 8a are related to the change in velocity (acceleration) in a short time; thus, the load can be characterised as the impact load.

The measured impulses in the time record are caused by the response of the lubrication film via a non-Newtonian nonlinear fluid effect. The time record in Figure 8a shows the impulse response of the EHDL. The measured signal responds as a dynamic system of nonlinear behaviour to a variable rate of radial loading of the bearing contact surfaces.

The physical–mechanical properties of the lubrication film are the source of the impulse response in the above-mentioned operating conditions when using a new defect-free bearing meeting all quality conditions. The lubrication film behaves as a viscous fluid, that is, a Newtonian fluid typically following Newton’s law of viscosity, with small velocity changes in the excitation force. Conversely, with large velocity changes in the excitation force, the response of the lubricating film is non-Newtonian. The lubrication film behaves as a solid-like material—that is, a non-Newtonian fluid that changes its viscosity or flow behaviour under stress. Such fluid is resistant to movement, and its behaviour significantly depends on the change of the applied force in time, which can be characterised as the impact load.

The conditions of the elasto-hydrodynamic contact are in a nonsteady state. Changing the operating conditions also changes the conditions of the elasto-hydrodynamic contact. Of course, the impact load is not the only factor that causes the impulse response. The non-Newtonian behaviour of the lubrication film and the subsequent impulse response of the elasto-hydrodynamic contact are caused by several factors. Further experiments were performed to determine the conditions under which the effect of the impulse response of the lubrication film is observed. The conditions were determined by experimental analyses and are presented in Table 3.

These impulse response factors may not be met at the same time. It is sufficient if the conditions of two or three factors are met or if any of the above factors is significantly more unfavourable than the limit values in Table 1.

### 3.1. Impulse Response for Different Rotor Bearings

**Figure 9:**
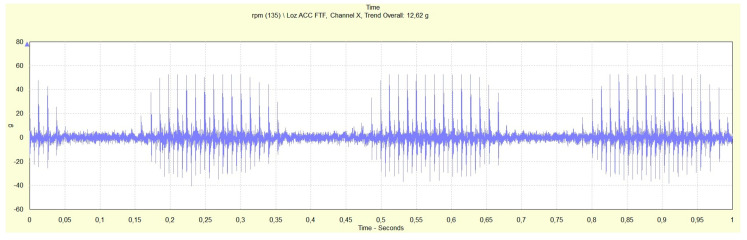
Impulse response (acceleration (g) vs. time (s)) for rotor bearing B (bearing heated by running), rotational speed of rotor bearing 135,000 rpm, drive pulleys rotational frequencies of 76.42 and 79.55 Hz.

**Figure 10:**
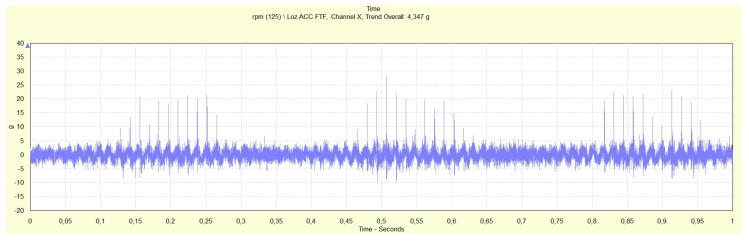
Impulse response (acceleration (g) vs. time (s)) for rotor bearing C, rotational speed of rotor bearing 125,000 rpm, drive pulleys rotational frequencies of 71.07 and 74.01 Hz.

Time records in Figure 9 and Figure 10 provide impulse responces of the EHDL in rotor bearings B and C (rotor bearing A is in Figure 8).

The lower rotational speed (125,000 rpm, rotor bearing C) caused the impulse of lower energy as the maximum acceleration of impulse is 28 g comparing to 62 g for higher rotational speed (135,000 rpm). In sections of time records without an impulse response of the EHDL, the conditions of impact load and thus fast radial deformation of lubrication film are not created, and the EHDL behaves as Newtonian fluid. In sections with an impulse response of EHDL, the opposite is true. Morever, Table 4 shows that the temperature slightly influences the impulse response of the EHDL (rotor bearing B).

The other results are summarized in following table.

### 3.2. Same Rotor Bearing with and without Impulse Response

**Figure 11:**
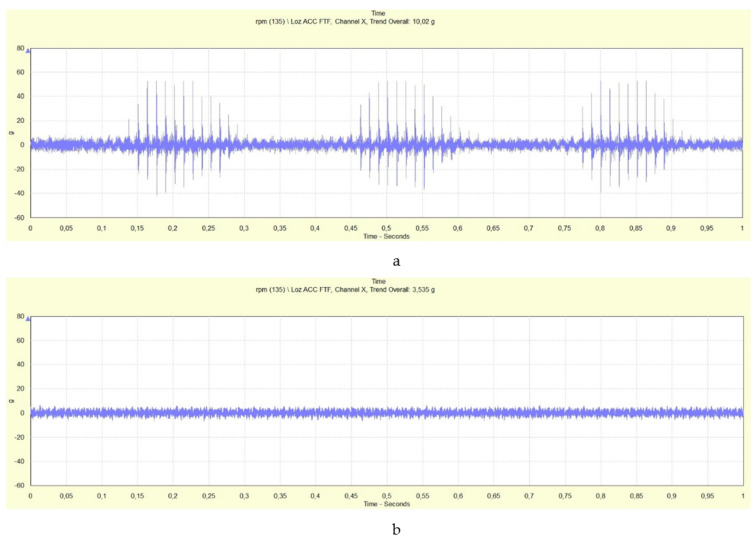
Time records of same bearing with (**a**) and without (**b**) the impulse responce.

The results mentioned above provide the impulse response of the EHDL evoked by beat loading, e.g., loading by two harmonic excitation forces close in rotational frequency resulting in a loading that a sinusoid is the envelope of the time record of the oscillations (beating load shown in Figure 7). Figure 11 presents the time records of rotor bearing with an impulse response at 135,000 rpm loaded by beat loading (Figure 11a) and the same rotor bearing at 135,000 rpm without an evoked impulse response of the EHDL (Figure 11b).

The sections in Figure 11a with the impulse response of the EHDL and without the response of the EHDL are visible in the time record. The section of the time record with an impulse response was caused only by the EHDL as the velocity of deformation of lubrication film was over the limit and the lubricant was reflected as non-Newtonian fluid in form of impulses. In Figure 11b, the EHDL did not respond by impulses, as there was no limit velocity (acceleration) in loading excitation, and subsequently, the “slow” deformation of lubrication film did not cause the impulse response.

## 4. Discussion

The high local energy impulse response of the EHDL causes the decomposition of the lubricant, which is a source of hydrogen and thus can embrittle the steel. Consequently, the bearing steel structure can be damaged in a short time (hours to weeks) by premature failures. This specific fatigue damage is called white etching crack (WEC) failure. The assumed initiation and propagation of WECs is described in more detail in [18]. The presence of WECs correlates with the applied cumulative energy. In our case, it is a high-energy impulse of the non-Newtonian effect of the lubrication film. The impulses documented in Figure 8 broke the bonds between carbon and hydrogen, which are the main components of the lubricant. Subsequently, hydrogen diffuses into the bearing steel, which causes hydrogen embrittlement of the steel, accelerating fatigue damage by up to 50 times [19,20]. In recent decades, WECs have become most commonly associated with failures of high-speed and intermediate-speed shaft bearings of wind turbine gearboxes [19].

Under the described conditions of elasto-hydrodynamic contact, bearing life decreases significantly to 5–10% [18] from a well-known static calculation: an empirical relationship for calculating bearing life *L*_10_ proposed by Palmgren in 1924 [21] and later (1947) developed by the Lundberg and Palmgren theory and equation.

## 5. Conclusions

The impulse responce of the EHDL is a universal effect resulting from its physical–mechanical properties. Until now, the majority of technical diagnosticians state that the impulse shape of the dynamic signal occurs in the case of damage to the raceways and rolling elements, in the case of low carrying capacity of the lubrication film and in case of a partial metal contact.

The experimental results prove that a rolling bearing as a dynamic system is a highly nonlinear system in certain modes of operation. Under certain physical conditions, the EHDL exhibits an impulse response to the periodic dynamic component of the load realised in a sinusoidal shape (beat cycles). The experiment simulates the operating mode in which the conditions under which the non-Newtonian behaviour of the lubricating layer and the subsequent impulse response occur. Such an operating mode can also occur with a new and undamaged bearing with complete oil friction without metal contact, as shown in this study. The impulses recorded in the presented measurement are not caused by defects in the raceways of the bearing or rolling elements but are generated by an EHDL under the described conditions. The impulse response of the lubricating layer is high frequency and high energy. This is the cause of premature fatigue damage to the bearing in the form of pitting. In addition, serious industrial accidents can occur repeatedly despite the repairs and replacement of bearings.

The presented effect of impulse response as a property of the EHDL develops even in various types of lubricant and various materials of bearing components. The impulse response of EHDL can be modified changing the types of lubricants and materials, but the occurrence of that response still occurs.

Further research should focus on the physical parameters of the dynamic system, as these affect the magnitude of the amplitude of the impulse response of the lubricating layer. Tests indicate that by modifying the chemical composition of the lubricant and adding specific advanced additives, it is possible to change the physical and dynamic properties of the EHDL system. Research into advanced bearing steels with optimised chemical composition and fine-grained microstructure after heat treatment shows great potential for increasing durability and extending the time to fracture–fatigue damage. Modern fine-grained bearing steels exhibit a significant increase in bearing life even under such difficult conditions; as mentioned in our study, it is possible to expect several times longer durability lifetimes than is achieved with the use of conventional bearing steels.

## Figures and Tables

**Table 1:** Materials.

Component	Material
Rolling balls	ceramics
Bearing shaft	fine-grained special steel
Cage	fibre-reinforced polyamide
Bearing bush	standard bearing steel
Lubrication	high-performance lubricating grease

**Table 2:** Parameters of raceways microgeometry quality.

Raceways	Textile Rotor Bearing	Standard Ball Bearing
Roughness *Ra* [μm]	0.02	0.08
Waviness (10–500 waves) [μm]	0.05	0.5
Roundness (2–10 waves) [μm]	0.16	1.0
Tolerance of surface profile [μm]	10	50

**Table 3:** Impulse response factors of the lubrication film.

Factor Number	Factor	Limit Value
1	rate of change of the applied force	>7 mm/s
2	static load	>1000 MPa
3	dynamic component of the applied force	in range > 3%
4	thickness of the lubrication film	<0.5 μm
5	microgeometry of contact surfaces	increased roughness (Ra > 0.1 μm), waviness, wear
6	kinematic slipping	>3‰

**Table 4:** Results for different rotor bearings.

Rotor Bearing	Running Speed of Rotor Bearing [rpm]	Rotational Frequency of Pulley 1 [Hz]	Rotational Frequency of Pulley 2 [Hz]	Max. Acceleration of Impulse [g]	*T*_B_ [s]
A	134,870	76.43	79.63	62	0.3125
B heated bearing	134,866	76.42	79.55	51	0.3195
B cold bearing (start)	134,866	76.42	79.55	53	0.3195
C	125,124	71.07	74.01	28	0.3401
